# Diagnostic accuracy of coronary opacification derived from coronary computed tomography angiography to detect ischemia: first validation versus single-photon emission computed tomography

**DOI:** 10.1186/s13550-017-0342-8

**Published:** 2017-11-25

**Authors:** Dominik C. Benz, Fran Mikulicic, Christoph Gräni, Marvin Grossmann, Andreas A. Giannopoulos, Michael Messerli, Catherine Gebhard, Oliver Gaemperli, Ronny R. Buechel, Philipp A. Kaufmann, Aju P. Pazhenkottil

**Affiliations:** Department of Nuclear Medicine, Cardiac Imaging, University Hospital Zurich, Ramistrasse 100, 8091 Zurich, Switzerland

**Keywords:** CT-derived functional parameters, CCO decrease, Corrected contrast opacification, Transluminal attenuation gradient, TAG

## Abstract

**Background:**

Estimation of functional relevance of a coronary stenosis by fractional flow reserve (FFR) from coronary computed tomography angiography (CCTA) has recently provided encouraging results. Due to its limited availability, the corrected contrast opacification (CCO) decrease and the transluminal attenuation gradient (TAG) were suggested as less complex alternatives. The aim of the present study was to assess the accuracy of CCO decrease and TAG to predict ischemia as assessed by single-photon emission computed tomography (SPECT) myocardial perfusion imaging (MPI).

**Results:**

This retrospective study included 72 patients who underwent hybrid CCTA/SPECT MPI with at least one coronary artery stenosis. Of 127 vessels with a coronary stenosis in CCTA, 38 (30%) were causing ischemia in its subtending myocardium. The area under the curve (AUC) for CCO decrease to predict ischemia was 0.707 with sensitivity, specificity, negative predictive value, positive predictive value, and accuracy of 74, 64, 85, 47, and 67%, respectively. For TAG, the AUC was 0.469.

**Conclusions:**

CCTA-derived CCO decrease but not TAG predicts ischemia in SPECT MPI. The negative predictive value of CCO decrease of 85% may confer clinical implications in the diagnostic work-up of patients with a coronary stenosis.

## Background

Coronary computed tomography angiography (CCTA) is a robust non-invasive tool to exclude coronary artery disease (CAD) [1, 2]. Conversely, its performance is moderate in assessing the functional relevance of a coronary stenosis [3]. Documenting evidence of functional relevance of a coronary stenosis is recommended prior to any revascularization procedure to improve outcome [4–6]. Hence, functional information should complement pure anatomic characterization of a coronary stenosis for appropriate clinical decision-making [7]. Cardiac hybrid imaging is a method to assess coronary anatomy and function at the same time by combining CCTA and single-photon emission computed tomography (SPECT) myocardial perfusion imaging (MPI). The added clinical and prognostic value of cardiac hybrid imaging has been demonstrated [8–11]. Despite these developments, there is growing interest in a single imaging modality that allows comprehensive morphological and functional assessment. Besides static and dynamic CT perfusion [12], recent studies have suggested that functional relevance of a coronary lesion can be estimated by fractional flow reserve (FFR) derived from CCTA (FFR_CT_) with high diagnostic accuracy [13] and positive impact on downstream resource utilization [14]. However, since the computation of FFR_CT_ is a cumbersome process with limited availability, faster and less complex alternative CT-derived parameters have evolved, such as the decrease in corrected contrast opacification (CCO) across a stenosis and the transluminal attenuation gradient (TAG) along a coronary vessel [15–18]. Despite the widespread adoption of SPECT MPI into clinical routine and its utility in appropriate clinical decision-making [19, 20], none of the previous studies investigating CCO decrease and TAG has used SPECT MPI as the standard of reference. Therefore, the present study aims at evaluating the diagnostic accuracy of CCO decrease and TAG in comparison to SPECT MPI.

## Methods

### Study population

The present retrospective study consists of 72 consecutive patients who underwent hybrid CCTA/SPECT MPI and had at least one coronary stenosis (i.e., luminal diameter narrowing ≥ 50% as assessed in CCTA). Exclusion criteria were history of coronary artery bypass graft (CABG) or stenting.

### CCTA acquisition and interpretation

On a stand-alone 64-slice CT scanner (LightSpeed VCT, GE Healthcare), patients underwent contrast-enhanced CCTA with prospective electrocardiography (ECG) triggering [21]. The following scanning parameters were applied: slice acquisition 64 × 0.625 mm, smallest X-ray window (only 75% of the RR-cycle), *z*-coverage value of 40 mm with an increment of 35 mm, gantry rotation time 350 ms, body mass index (BMI) adapted tube voltage (100 kV, BMI < 25 kg/m^2^; 120 kV, BMI ≥ 25 kg/m^2^), and effective tube-current (450 mA, BMI < 22.5 kg/m^2^; 500 mA, BMI 22.5–25 kg/m^2^; 550 mA, BMI 25–27.5 kg/m^2^; 600 mA, BMI 27.5–30 kg/m^2^; 650 mA, BMI > 30 kg/m^2^). Bolus tracking was performed with a region of interest (ROI) placed into the ascending aorta, and image acquisition was started 4 s after the signal density reached a predefined threshold of 120 Hounsfield units (HU). In order to achieve a target heart rate < 65 bpm, intravenous metoprolol (5–20 mg) was administered prior to the CCTA examination if necessary. Furthermore, all patients received 2.5 mg sublingual isosorbiddinitrate 2 min prior to the scan.

**Fig. 1:**
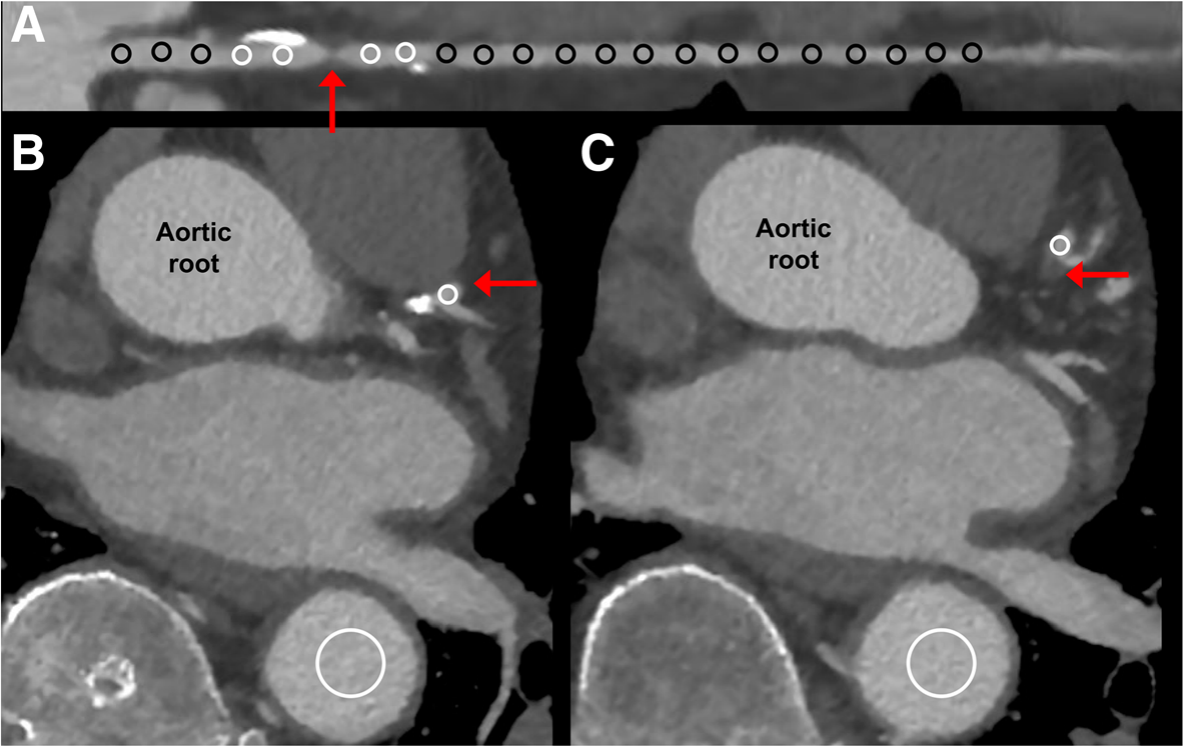
Example of CCO decrease and TAG assessment. A soft plaque with high-grade stenosis is located in the proximal LAD (**a**, red arrow). Two coronary ROIs (**a**, small white circles) are placed proximal (**b**) and distal of a lesion (**c**). Corrected contrast opacification (CCO) is calculated as the ratio of mean attenuation in the coronary ROI over the ROI in the descending aorta (large white circles). Using the lower of the two values, the difference between the proximal (**b**) and distal (**c**) was defined as CCO decrease. The transluminal attenuation gradient (TAG) was measured by ROIs at 5-mm intervals from the ostium to the distal segment where the vessel cross-sectional area decreases below 2 mm^2^ (**a**, small black circles) and defined as the linear regression coefficient between intraluminal attenuation and distance from the ostium

CCTA images were analyzed by consensus of two experienced readers with regard to morphologically significant lesions (≥ 50%). CCO decrease was measured for each stenosis as previously described [17]. In brief, a region of interest (ROI) with a diameter of 1 mm was placed in the center of the coronary lumen and a ROI with a diameter of 10 mm was placed in the descending aorta (on the same axial slice). CCO was calculated as the ratio of mean attenuation in the coronary ROI over the aorta ROI. CCO was measured twice within 2 cm proximal and distal of a lesion. The difference between the proximal and the distal CCO (using the lower of the two CCO values) was defined as CCO decrease (Fig. 1). If multiple lesions were present within one vessel, only the highest CCO decrease was used for further calculation of diagnostic accuracy.

TAG was measured in each major vessel with a coronary stenosis while side-branch vessels were not included in TAG assessment as previously described [22]. The luminal centerline was determined, and perpendicular cross-sectional images were reconstructed. At 5-mm intervals from the ostium to the distal level where the vessel cross-sectional area decreases below 2.0 mm^2^, a ROI with a diameter of 1 mm was manually positioned in the luminal center and mean HU were determined. In order to maintain linearity of the gradient, excessively calcified coronary segments were excluded as previously reported [22]. TAG was defined as the linear regression coefficient between intraluminal attenuation in HU and length from the ostium (Fig. 1).

### SPECT MPI acquisition and interpretation

A 1-day ECG-gated stress/rest protocol was used with pharmacologic stress induced by infusion of adenosine at a standard rate of 140 μg/kg/min, and a BMI-adapted dose of 250 to 350 MBq 99mTc-tetrofosmin was injected 3 min into the pharmacologic stress [23]. After a delay of 60 min, stress images were acquired during 15 min. Immediately thereafter, a threefold higher dose 99mTc-tetrofosmin was administered and rest images were acquired during 15 min. In all patients, gated images were acquired with a dual-head camera (Millenium VG and Hawkeye or Ventri, both GE Healthcare) using standard acquisition parameters and X-ray-based attenuation correction. A commercially available software package (Cedars QGS/QPS, Los Angeles, CA, USA) was used for image analysis. Identification of ischemia and allocation to its corresponding coronary vessel was performed as previously reported [10]. In brief, SPECT myocardial tomograms were split into 20 segments for each patient. These segments were analyzed by consensus of two experienced readers using the following five-point scoring system: 0, normal; 1, equivocal; 2, moderate; 3, severe reduction of radioisotope uptake; and 4, absence of detectable tracer in a segment. A scan was scored as abnormal if two or more segments had stress scores ≥ 2. A reversible perfusion defect was categorized as one in which a stress defect was associated with a rest score ≤ 1 or a stress defect score of ≥ 4 with a rest score of 2.

### Statistical analysis

Continuous variables are expressed as mean ± standard deviation (SD) or as median with interquartile range (IQR) if not normally distributed. Categorical variables are expressed as frequencies or percentages. Kolmogorov-Smirnov test was applied to assess normal distribution. *P* values for categorical variables were calculated by the chi-square test and for continuous variables by Mann-Whitney *U* test or Kruskal-Wallis test. We took into account the repeated structure of the measures and the hierarchical data structure (i.e., the fact that the segments and vessels were clusters of observations in the patients). Although generalized linear mixed modeling would be an excellent alternative, such analysis was restricted by the data structure (complexity due to multiple intrinsic hemodynamic and scan parameters) of the present study [24]. Receiver-operating characteristics (ROC) curve analysis was plotted to illustrate the performance of CCO decrease and TAG to diagnose ischemia. Youden’s index was calculated to define the optimal threshold. Sensitivity, specificity, positive predictive value, negative predictive value, and accuracy of CCO decrease and TAG were assessed on a per-vessel basis, in case the ROC curve analysis was statistically significant. A *p* value < 0.05 was considered statistically significant. SPSS 20.0 (IBM Corporation, Armonk, NY) was used for analysis.

## Results

### Study population

The baseline characteristics of the study population are summarized in Table 1. The 72 patients had 183 coronary lesions in 127 vessels (108 major vessels and 19 side-branch vessels).

**Table 1:** Baseline characteristics

Male gender, *n* (%)	54 (75)
Age (years)	63 ± 10
Imaging heart rate (bpm)	57 ± 7
Body mass index (kg/m^2^)	27 ± 4
Cardiovascular risk factors, *n* (%)	
Smoking	28 (39)
Diabetes	9 (13)
Hypertension	45 (63)
Dyslipidaemia	40 (56)
Family history of CAD	25 (35)
Median Agatston score (IQR)	564 (175–1368)
Reason referral, *n* (%)	
Pre-operative evaluation, equivocal/abnormal stress test	15 (21)
Atypical chest pain	30 (42)
Typical angina pectoris	15 (21)
Dyspnoea	12 (17)
Cardiac history, *n* (%)	
Previous myocardial infarction	3 (4)
Clinical pre-test probability (%)	51 ± 20

Mean ± standard deviation, if not otherwise specified

### CCTA and SPECT findings

**Fig. 2:**
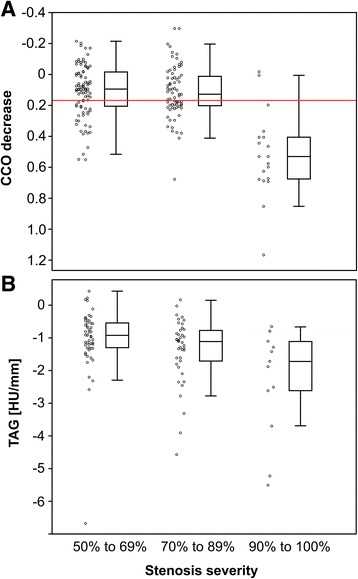
CCO decrease and TAG according to stenosis severity. Box-and-whisker plot showing corrected contrast opacification (CCO) decrease (**a**) and TAG (**b**) of all lesions in the categories of 50 to 69, 70 to 89, and 90 to 100% diameter stenosis. The red horizontal line corresponds to the cutoff value for an abnormal CCO decrease as used in the present study (CCO decrease > 0.168). The boxes represent the interquartile range (IQR) and the dark line in the middle is the median. The whiskers are defined as 1.5 times the IQR

CCO decrease was successfully assessed in all 183 coronary lesions of the 127 vessels (100%). Distribution of CCO decrease according to stenosis severity is illustrated in Fig. 2a. Median CCO decrease was 0.094 (IQR, − 0.017 to 0.209), 0.128 (IQR, 0.005 to 0.205), and 0.530 (IQR, 0.405 to 0.678) for lesions with 50–69, 70–89, and 90–100% stenosis severity, respectively. While CCO decrease was significantly different across stenosis severities (*p* < 0.001), comparison between lesions of 50–69% and 70–89% stenosis severity revealed no significant difference (*p* = 0.470).

TAG was successfully assessed in 106 major vessels with a coronary stenosis (98%). Distribution of TAG according to stenosis severity is illustrated in Fig. 2b. Median TAG was − 0.9 (IQR, − 1.3 to − 0.6), − 1.1 (IQR, − 1.8 to − 0.8), and − 1.7 (IQR, − 3.2 to − 1.0) for vessels with the most severe lesion of 50–69, 70–89, or 90–100% stenosis severity. While TAG was significantly different across stenosis severities (*p* < 0.05), comparison between lesions of 50–69 and 70–89% stenosis severity only trended towards a difference (*p* = 0.069).

**Fig. 3:**
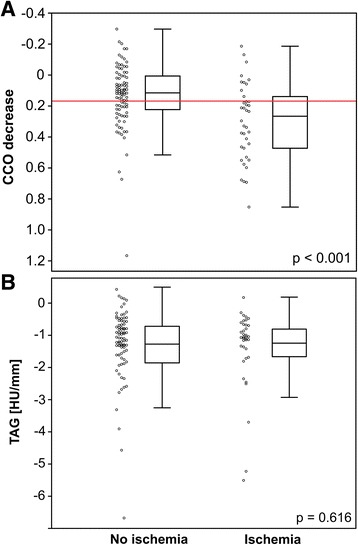
CCO decrease and TAG in ischemic and non-ischemic myocardium. Box-and-whisker plot showing CCO decrease (**a**) and TAG (**b**) in ischemic and non-ischemic myocardium. The red horizontal line corresponds to the cutoff value for an abnormal CCO decrease as defined in the present study (CCO decrease > 0.168). The boxes represent the interquartile range (IQR) and the dark line in the middle is the median. The whiskers are defined as 1.5 times the IQR

Of 127 vessels with a coronary stenosis, 38 vessels (including four side-branch vessels) (30%) were subtended by ischemic myocardium. In these vessels, median CCO decrease was significantly higher compared to vessels subtended by non-ischemic myocardium (0.266 vs. 0.115; *p* < 0.001; Fig. 3a). In contrast, median TAG remained unchanged in vessels subtended by ischemic myocardium (− 1.1 HU/mm vs. − 1.1 HU/mm; *p* = 0.616; Fig. 3b).

### Diagnostic accuracy of CCO decrease and TAG

**Fig. 4:**
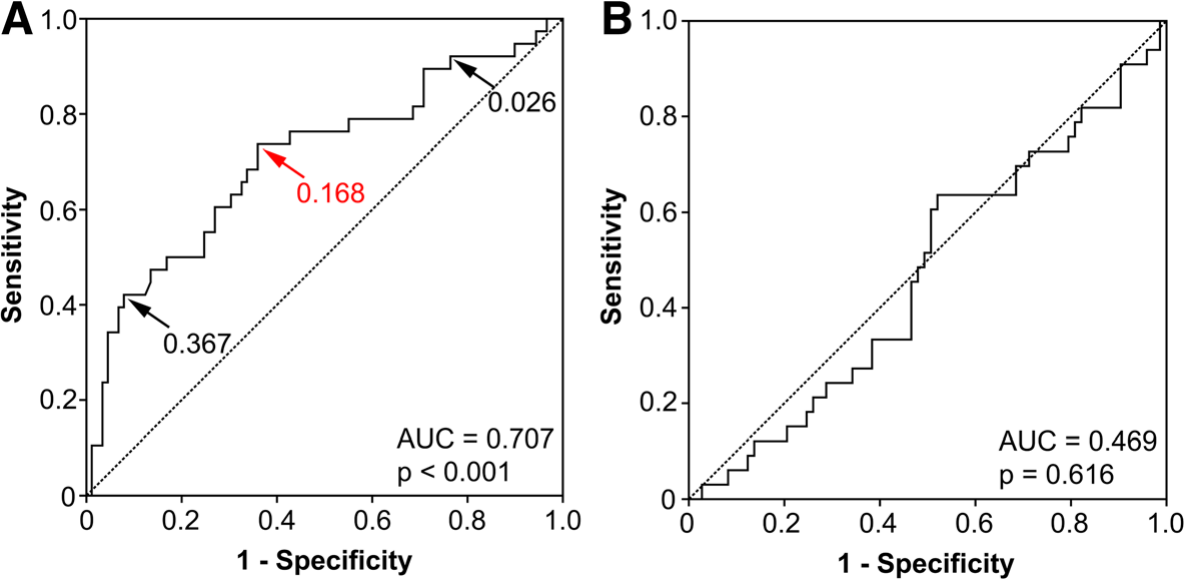
ROC curve of CCO decrease and TAG. While CCO decrease (**a**) significantly predicted ischemia with an AUC of 0.707 (*p* < 0.001) in the ROC curve analysis, TAG (**b**) did not (indicated by an AUC of 0.469; *p* = 0.616). The arrows in panel **a** indicate the cutoff value for an abnormal CCO. By adjusting the cutoff value from 0.168 (as defined in the present study) to 0.026, sensitivity would increase to 90% (at the cost of reducing specificity to 29%). On the contrary, a cutoff value at 0.367 would increase specificity to 90% (but decrease sensitivity to 42%)

The ROC curve analysis resulted in an area under the curve (AUC) of 0.707 (*p* < 0.001) for CCO decrease to predict ischemia (Fig. 4a). The optimal threshold of CCO decrease was defined at 0.168. Implementing the latter, an abnormal CCO decrease correctly detected ischemia in 28 of 38 vessels (sensitivity, 74%; 95% CI 57 to 87%) and correctly ruled out ischemia in 57 of 89 (specificity, 64%; 95% CI 53 to 74%) vessels. This resulted in a negative predictive value, positive predictive value, and accuracy of 85% (95% CI 74 to 93%), 47% (95% CI 34 to 60%), and 67%, respectively. After exclusion of lesions with 90–100% stenosis severity, the ROC curve analysis resulted in an AUC of 0.649 (*p* < 0.05). The optimal threshold of CCO decrease was unchanged. It correctly detected ischemia in 18 of 28 vessels (64%) and correctly ruled out ischemia in 55 of 82 vessels (67%). This resulted in a sensitivity, specificity, negative predictive value, positive predictive value, and accuracy of 64% (95% CI 44 to 81%), 67% (95% CI 56 to 77%), 85% (95% CI 74 to 92%), 40% (95% CI 26 to 56%), and 66%, respectively.

The ROC curve revealed an AUC of 0.469 (*p* = 0.616; Fig. 4b), indicating that TAG is no predictor for ischemia.

## Discussion

The present study demonstrates that an abnormal CCO decrease has an encouraging negative predictive value to exclude functionally relevant lesions conferring potential implication in the diagnostic work-up of patients with low pre-test probability for CAD which is the population benefiting most from CCTA. While median CCO decrease was significantly higher in vessels subtended by ischemic myocardium, TAG remained unchanged. Consequently, TAG did not predict ischemia in patients with a coronary stenosis.

While previous studies have reported on the diagnostic value of CCO decrease and TAG in comparison to thrombolysis in myocardial infarction (TIMI) flow, cardiac magnetic resonance imaging, and invasive FFR, our results are the first to compare their performance in the assessment of the functional relevance of a coronary stenosis with SPECT MPI. Considering the fact that invasive FFR was originally validated against SPECT MPI [25] and that there exists substantial mismatch between invasive FFR and SPECT [26], the direct comparison between CCO decrease and SPECT MPI extends our understanding of CCO decrease as a clinical tool. The diagnostic accuracy of CCO decrease in the present study is lower than that in the previous studies despite the comparable baseline characteristics [15–17]. Previous studies included substantially smaller study populations [15, 16] or used other techniques as a standard of reference (TIMI flow in a previous report [17] vs. SPECT MPI in the present study). Comparing CCO decrease to PET MPI, the ultimate gold standard for non-invasive assessment of myocardial blood flow has resulted in a diagnostic accuracy of 70% and a cutoff for an abnormal CCO decrease at 0.166 [18], matching well with the present study. Conversely, the literature is inconsistent concerning the diagnostic accuracy of TAG. While several reports have demonstrated an incremental value to diagnose functionally relevant lesions over anatomic CCTA findings [27–29], studies without single-beat acquisition rarely revealed a diagnostic benefit for TAG [30, 31]. Overall, the finding that differences in resting CCTA contrast densities may predict functional relevance is astounding, particularly in view of the evidence that an expected reduction in resting flow only occurs in lesions with a stenosis degree of 90% and above [32, 33]. The distribution of stenosis severity across the patient population, therefore, is key to the interpretation of previous studies and could potentially contribute to the inconsistencies in the literature. In the present study, however, if the analysis was limited to lesions below 90% diameter stenosis, diagnostic accuracy did only change marginally. Thus, CCO decrease seems to add moderate but significant value to the evaluation of intermediate lesions. This finding—that functional relevance of an anatomic coronary lesion can be assessed without requiring any hyperemic stress—is in line with previous studies measuring instantaneous wave-free ratio (iFR) [34]. Interestingly, iFR estimates coronary pressure during diastole when resting resistance is lowest—a timing interval similar to the one used by prospectively ECG-triggered CCTA [35].

A large body of evidence has documented that the presence of a functionally relevant stenosis impairs outcome, regardless by which modality the latter was assessed [4, 10, 19, 36, 37]. As a consequence, physiological assessment of stenosis relevance is critical in decision-making for appropriate treatment strategy as both revascularization of non-relevant lesions [6] and deferral from revascularization of hemodynamically relevant lesions may lead to a less favorable outcome [4, 5]. Although current guidelines mandate functional assessment for evidence-based revascularization [7], both non-invasive stress testing before invasive coronary angiography and FFR before percutaneous coronary intervention remain underused in daily practice [38, 39]. By its ease of use at no additional costs, CCO decrease may increase the probability for a comprehensive anatomic and functional non-invasive assessment before the patient is referred to invasive coronary angiography. Due to its moderate diagnostic accuracy, CCO decrease may not replace MPI, but it could potentially be positioned as a gatekeeper after CCTA to allocate further non-invasive diagnostic work-up. Although any test added to CCTA should ideally feature high positive predictive value, this does not apply to CCO decrease [16]. However, its encouraging negative predictive value and, as recently demonstrated, its added prognostic value [40] indicates a role to individualize clinical workflow: CCTA identifies a stenosis but if CCO decrease is normal, patients might be deferred from further MPI [41]. In contrast, if CCO decrease is abnormal over a stenosis, further non-invasive testing should be considered. In order to minimize the number of underdiagnosed patients, the cutoff value of an abnormal CCO decrease could be adjusted from 0.168 to lower values, e.g., a cutoff value at 0.026 would increase sensitivity to 90% (Fig. 4a). Through this workflow, downstream resource utilization might be influenced in a cost-effective manner. This may hold true in particular for the population which is typically referred for CCTA, namely patients with low to intermediate pre-test probability.

We acknowledge the following limitations. First, the present study is a retrospective single-center study and has all of the inherent limitations of that study design. Second, the threshold for CCO decrease as defined in the present study should be extrapolated with caution to different patient populations since differences not only in baseline characteristics but also in scan protocol and post-processing might influence analysis of CT images [42, 43]. Third, it cannot be excluded that the lack of a diagnostic value of TAG was due to the inferior performance of TAG with 64-slice scanners [27, 30] compared to wide-volume scanners [28, 44]. With the wider distribution of 256-slice CT scanners [45], future studies may assess the diagnostic value of TAG without this limitation.

## Conclusions

CCTA-derived CCO decrease but not TAG predicts ischemia in SPECT MPI. The negative predictive value of CCO decrease of 85% may confer clinical implications in the diagnostic work-up of patients with a coronary stenosis.

## Abbreviations

AUC: Area under the curve
CABG: Coronary artery bypass graft
CCO: Corrected contrast opacification
CCTA: Coronary computed tomography angiography
ECG: Electrocardiography
FFR: Fractional flow reserve
HU: Hounsfield units
iFR: Instantaneous wave-free ratio
MPI: Myocardial perfusion imaging
ROC: Receiver-operating characteristics
ROI: Region of interest
SPECT: Single-photon emission computed tomography
TAG: Transluminal attenuation gradient

## References

[1] Budoff MJ, Dowe D, Jollis JG, Gitter M, Sutherland J, Halamert E (2008). Diagnostic performance of 64-multidetector row coronary computed tomographic angiography for evaluation of coronary artery stenosis in individuals without known coronary artery disease: results from the prospective multicenter ACCURACY (Assessment by Coronary Computed Tomographic Angiography of Individuals Undergoing Invasive Coronary Angiography) trial. J Am Coll Cardiol.

[2] Benz DC, Fuchs TA, Gräni C, Studer Bruengger AA, Clerc OF, Mikulicic F, et al. Head-to-head comparison of adaptive statistical and model-based iterative reconstruction algorithms for submillisievert coronary CT angiography. Eur Heart J Cardiovasc Imaging. 2017; doi:10.1093/ehjci/jex008. 10.1093/ehjci/jex008 28200212

[3] Gaemperli O, Husmann L, Schepis T, Koepfli P, Valenta I, Jenni W (2009). Coronary CT angiography and myocardial perfusion imaging to detect flow-limiting stenoses: a potential gatekeeper for coronary revascularization?. Eur Heart J.

[4] De Bruyne B, Pijls NH, Kalesan B, Barbato E, Tonino PA, Piroth Z (2012). Fractional flow reserve-guided PCI versus medical therapy in stable coronary disease. N Engl J Med.

[5] De Bruyne B, Fearon WF, Pijls NH, Barbato E, Tonino P, Piroth Z (2014). Fractional flow reserve-guided PCI for stable coronary artery disease. N Engl J Med.

[6] Tonino PA, De Bruyne B, Pijls NH, Siebert U, Ikeno F, van’t Veer M (2009). Fractional flow reserve versus angiography for guiding percutaneous coronary intervention. N Engl J Med.

[7] Windecker S, Kolh P, Alfonso F, Collet JP, Cremer J, Falk V (2014). 2014 ESC/EACTS Guidelines on myocardial revascularization: the task force on myocardial revascularization of the European Society of Cardiology (ESC) and the European Association for Cardio-Thoracic Surgery (EACTS) developed with the special contribution of the European Association of Percutaneous Cardiovascular Interventions (EAPCI). Eur Heart J.

[8] Gaemperli O, Schepis T, Valenta I, Husmann L, Scheffel H, Duerst V (2007). Cardiac image fusion from stand-alone SPECT and CT: clinical experience. J Nucl Med.

[9] Liga R, Vontobel J, Rovai D, Marinelli M, Caselli C, Pietila M (2016). Multicentre multi-device hybrid imaging study of coronary artery disease: results from the EValuation of INtegrated Cardiac Imaging for the Detection and Characterization of Ischaemic Heart Disease (EVINCI) hybrid imaging population. Eur Heart J Cardiovasc Imaging.

[10] Pazhenkottil AP, Nkoulou RN, Ghadri JR, Herzog BA, Buechel RR, Kuest SM (2011). Prognostic value of cardiac hybrid imaging integrating single-photon emission computed tomography with coronary computed tomography angiography. Eur Heart J.

[11] Benz DC, Templin C, Kaufmann PA, Buechel RR (2015). Ultra-low-dose hybrid single photon emission computed tomography and coronary computed tomography angiography: a comprehensive and non-invasive diagnostic workup of suspected coronary artery disease. Eur Heart J.

[12] Danad I, Szymonifka J, Schulman-Marcus J, Min JK (2016). Static and dynamic assessment of myocardial perfusion by computed tomography. Eur Heart J Cardiovasc Imaging.

[13] Norgaard BL, Leipsic J, Gaur S, Seneviratne S, Ko BS, Ito H (2014). Diagnostic performance of noninvasive fractional flow reserve derived from coronary computed tomography angiography in suspected coronary artery disease: the NXT trial (analysis of coronary blood flow using CT angiography: next steps). J Am Coll Cardiol.

[14] Douglas PS, Pontone G, Hlatky MA, Patel MR, Norgaard BL, Byrne RA (2015). Clinical outcomes of fractional flow reserve by computed tomographic angiography-guided diagnostic strategies vs. usual care in patients with suspected coronary artery disease: the prospective longitudinal trial of FFRCT: outcome and resource impacts study. Eur Heart J.

[15] den Dekker MA, Pelgrim GJ, Pundziute G, van den Heuvel ER, Oudkerk M, Vliegenthart R (2015). Hemodynamic significance of coronary stenosis by vessel attenuation measurement on CT compared with adenosine perfusion MRI. Eur J Radiol.

[16] Wang R, Renker M, Schoepf UJ, Wichmann JL, Fuller SR, Rier JD (2015). Diagnostic value of quantitative stenosis predictors with coronary CT angiography compared to invasive fractional flow reserve. Eur J Radiol.

[17] Chow BJ, Kass M, Gagne O, Chen L, Yam Y, Dick A (2011). Can differences in corrected coronary opacification measured with computed tomography predict resting coronary artery flow?. J Am Coll Cardiol.

[18] Benz DC, Gräni C, Ferro P, Neumeier L, Messerli M, Possner M, et al. Corrected coronary opacification decrease from coronary computed tomography angiography: validation with quantitative 13N-ammonia positron emission tomography. J Nucl Cardiol. 2017; doi:10.1007/s12350-017-0980-2. 10.1007/s12350-017-0980-2 28685251

[19] Hachamovitch R, Hayes SW, Friedman JD, Cohen I, Berman DS (2003). Comparison of the short-term survival benefit associated with revascularization compared with medical therapy in patients with no prior coronary artery disease undergoing stress myocardial perfusion single photon emission computed tomography. Circulation.

[20] Shaw LJ, Weintraub WS, Maron DJ, Hartigan PM, Hachamovitch R, Min JK (2012). Baseline stress myocardial perfusion imaging results and outcomes in patients with stable ischemic heart disease randomized to optimal medical therapy with or without percutaneous coronary intervention. Am Heart J.

[21] Husmann L, Valenta I, Gaemperli O, Adda O, Treyer V, Wyss CA (2008). Feasibility of low-dose coronary CT angiography: first experience with prospective ECG-gating. Eur Heart J.

[22] Stuijfzand WJ, Danad I, Raijmakers PG, Marcu CB, Heymans MW, van Kuijk CC (2014). Additional value of transluminal attenuation gradient in CT angiography to predict hemodynamic significance of coronary artery stenosis. JACC Cardiovasc Imaging.

[23] Pazhenkottil AP, Ghadri JR, Nkoulou RN, Wolfrum M, Buechel RR, Küest SM (2011). Improved outcome prediction by SPECT myocardial perfusion imaging after CT attenuation correction. J Nucl Med.

[24] Heck RHTS, Tabata LN (2012). Multilevel modeling of categorical outcomes using IBM SPSS.

[25] Pijls NH, De Bruyne B, Peels K, Van Der Voort PH, Bonnier HJ, Bartunek JKJJ (1996). Measurement of fractional flow reserve to assess the functional severity of coronary-artery stenoses. N Engl J Med.

[26] Melikian N, De Bondt P, Tonino P, De Winter O, Wyffels E, Bartunek J (2010). Fractional flow reserve and myocardial perfusion imaging in patients with angiographic multivessel coronary artery disease. JACC Cardiovasc Interv.

[27] Choi JH, Koo BK, Yoon YE, Min JK, Song YB, Hahn JY (2012). Diagnostic performance of intracoronary gradient-based methods by coronary computed tomography angiography for the evaluation of physiologically significant coronary artery stenoses: a validation study with fractional flow reserve. Eur Heart J Cardiovasc Imaging.

[28] Wong DT, Ko BS, Cameron JD, Nerlekar N, Leung MC, Malaiapan Y (2013). Transluminal attenuation gradient in coronary computed tomography angiography is a novel noninvasive approach to the identification of functionally significant coronary artery stenosis: a comparison with fractional flow reserve. J Am Coll Cardiol.

[29] Choi JH, Min JK, Labounty TM, Lin FY, Mendoza DD, Shin DH (2011). Intracoronary transluminal attenuation gradient in coronary CT angiography for determining coronary artery stenosis. JACC Cardiovasc Imaging.

[30] Yoon YE, Choi JH, Kim JH, Park KW, Doh JH, Kim YJ (2012). Noninvasive diagnosis of ischemia-causing coronary stenosis using CT angiography: diagnostic value of transluminal attenuation gradient and fractional flow reserve computed from coronary CT angiography compared to invasively measured fractional flow reserve. JACC Cardiovasc Imaging.

[31] Tesche C, De Cecco CN, Caruso D, Baumann S, Renker M, Mangold S (2016). Coronary CT angiography derived morphological and functional quantitative plaque markers correlated with invasive fractional flow reserve for detecting hemodynamically significant stenosis. J Cardiovasc Comput Tomogr.

[32] Klocke FJ (1983). Measurements of coronary blood flow and degree of stenosis: current clinical implications and continuing uncertainties. J Am Coll Cardiol.

[33] Gould KL, Lipscomb K, Hamilton GW (1974). Physiologic basis for assessing critical coronary stenosis. Instantaneous flow response and regional distribution during coronary hyperemia as measures of coronary flow reserve. Am J Cardiol.

[34] Jeremias A, Maehara A, Genereux P, Asrress KN, Berry C, De Bruyne B (2014). Multicenter core laboratory comparison of the instantaneous wave-free ratio and resting Pd/Pa with fractional flow reserve: the RESOLVE study. J Am Coll Cardiol.

[35] Clerc OF, Kaufmann BP, Possner M, Liga R, Vontobel J, Mikulicic F, et al. Long-term prognostic performance of low-dose coronary computed tomography angiography with prospective electrocardiogram triggering. Eur Radiol. 2017; doi:10.1007/s00330-017-4849-1. 10.1007/s00330-017-4849-1 28500370

[36] Jahnke C, Nagel E, Gebker R, Kokocinski T, Kelle S, Manka R (2007). Prognostic value of cardiac magnetic resonance stress tests: adenosine stress perfusion and dobutamine stress wall motion imaging. Circulation.

[37] Murthy VL, Naya M, Foster CR, Hainer J, Gaber M, Di Carli G (2011). Improved cardiac risk assessment with noninvasive measures of coronary flow reserve. Circulation.

[38] Buechel RR, Kaufmann BA, Tobler D, Wild D, Zellweger MJ (2015). Non-invasive nuclear myocardial perfusion imaging improves the diagnostic yield of invasive coronary angiography. Eur Heart J Cardiovasc Imaging.

[39] Harle T, Zeymer U, Hochadel M, Zahn R, Kerber S, Zrenner B (2017). Real-world use of fractional flow reserve in Germany: results of the prospective ALKK coronary angiography and PCI registry. Clin Res Cardiol.

[40] Benz DC, Mikulicic F, Gräni C, Moret D, Possner M, Clerc OF, et al. Long-term outcome prediction by functional parameters derived from coronary computed tomography angiography. Int J Cardiol. 2017; doi:10.1016/j.ijcard.2017.05.083. 10.1016/j.ijcard.2017.05.083 28592383

[41] Cademartiri F, Maffei E. Anatomy and physiology in coronary artery disease imaging. J Nucl Cardiol. 2017; doi:10.1007/s12350-017-1033-6. 10.1007/s12350-017-1033-6 28815422

[42] Benz DC, Gräni C, Mikulicic F, Vontobel J, Fuchs TA, Possner M (2016). Adaptive statistical iterative reconstruction-V: impact on image quality in ultralow-dose coronary computed tomography angiography. J Comput Assist Tomogr.

[43] Benz DC, Gräni C, Hirt Moch B, Mikulicic F, Vontobel J, Fuchs TA (2017). A low-dose and an ultra-low-dose contrast agent protocol for coronary CT angiography in a clinical setting: quantitative and qualitative comparison to a standard dose protocol. Br J Radiol.

[44] Ko BS, Wong DT, Norgaard BL, Leong DP, Cameron JD, Gaur S (2016). Diagnostic performance of transluminal attenuation gradient and noninvasive fractional flow reserve derived from 320-detector row CT angiography to diagnose hemodynamically significant coronary stenosis: an NXT substudy. Radiology.

[45] Benz DC, Grani C, Hirt Moch B, Mikulicic F, Vontobel J, Fuchs TA (2016). Minimized radiation and contrast agent exposure for coronary computed tomography angiography: first clinical experience on a latest generation 256-slice scanner. Acad Radiol.

